# Structured training pathway and proctoring; multicenter results of the implementation of transanal total mesorectal excision (TaTME) in the Netherlands

**DOI:** 10.1007/s00464-019-06750-w

**Published:** 2019-03-19

**Authors:** M. Veltcamp Helbach, S. E. van Oostendorp, T. W. A. Koedam, J. J. Knol, H. B. A. C. Stockmann, S. J. Oosterling, R. C. L. M. Vuylsteke, E. J. R. de Graaf, P. G. Doornebosch, R. Hompes, H. J. Bonjer, C. Sietses, J. B. Tuynman

**Affiliations:** 1 Department of Surgery, Amsterdam UMC, Location VUmc, De Boelelaan 1117, 1081 HV Amsterdam, The Netherlands; 2 Department of Surgery, Jessa Hospital, Hasselt and Herk-de-Stad, Belgium; 3 Department of Surgery, Spaarne Gasthuis, Haarlem and Hoofddorp, The Netherlands; 4 Department of Surgery, IJsselland Hospital, Cappelle a/d Ijssel, The Netherlands; 5 Department of Surgery, Amsterdam UMC, Location AMC, Amsterdam, The Netherlands; 6 Department of Surgery, Gelderse Vallei Hospital, Ede, The Netherlands

**Keywords:** TaTME, TAMIS, Rectal cancer, Proctoring, Training

## Abstract

**Background:**

Transanal total mesorectal excision **(**TaTME) is a new complex technique with potential to improve the quality of surgical mesorectal excision for patients with mid and low rectal cancer. The procedure is technically challenging and has shown to be associated with a relative long learning curve which might hamper widespread adoption. Therefore, a national structured training pathway for TaTME has been set up in the Netherlands to allow safe implementation. The aim of this study was to monitor safety and efficacy of the training program with 12 centers.

**Methods:**

Short-term outcomes of the first ten TaTME procedures were evaluated in 12 participating centers in the Netherlands within the national structured training pathway. Consecutive patients operated during and after the proctoring program for rectal carcinoma with curative intent were included. Primary outcome was the incidence of intraoperative complications, secondary outcomes included postoperative complications and pathological outcomes.

**Results:**

In October 2018, 12 hospitals completed the training program and from each center the first 10 patients were included for evaluation. Intraoperative complications occurred in 4.9% of the cases. The clinicopathological outcome reported 100% for complete or nearly complete specimen, 100% negative distal resection margin, and the circumferential resection margin was positive in 5.0% of patients. Overall postoperative complication rate was 45.0%, with 19.2% Clavien–Dindo ≥ III and an anastomotic leak rate of 17.3%.

**Conclusions:**

This study shows that the nationwide structured training program for TaTME delivers safe implementation of TaTME in terms of intraoperative and pathology outcomes within the first ten consecutive cases in each center. However, postoperative morbidity is substantial even within a structured training pathway and surgeons should be aware of the learning curve of this new technique.

Transanal total mesorectal excision (TaTME) has been proposed as a potentially better alternative to the laparoscopic standard TME for mid and distal rectal cancer [1–4]. The bottom-up approach for the deep pelvic region increases exposure and facilitates the distal mesorectal excision. Especially those difficult cases such as obese patients, low rectal cancer, and (male) patients with a narrow pelvis seem to benefit from this approach [5, 6]. TaTME has been introduced in 2010 and has had enthusiastic uptake throughout the world [7, 8]. Current evidence from non-randomized studies shows that the TaTME technique for mid and low rectal cancer has similar short-term clinical outcomes compared to laparoscopic TME (Lap TME) in terms of complications including anastomotic leakage, margin involvement, and specimen quality. Especially the conversion rate seems to benefit from TaTME compared to Lap TME (2% versus 12%) as shown in both registry and systematic reviews [8, 9]. The circumferential resection margin (CRM) involvement after TaTME has shown to be slightly lower as observed in conventional or robotic TME in pooled analysis by weighted averages: 4.6% versus 7.9% versus 5.1%, respectively [8–10]. However, direct comparison in large trials has yet to confirm this difference and demonstrate oncological safety. Within the international TaTME registry, a relative high percentage of intraoperative and postoperative complications has been presented including an anastomotic failure rate of 15.7% [9, 11]. Since the TaTME has only recently been introduced, a learning curve is probably partly a reason for the relative high morbidity rate. TaTME seems technically demanding because of the required single port surgery skills and due to a different approach to the anatomy: down-to-up [12]. The traditional landmarks are missing and surgeons inexperienced in this technique may encounter TaTME-specific related complications. A well-trained TaTME surgeon might show benefits, but for those less trained and capable surgery by TaTME will probably result in worse short- and long-term outcomes. A meta-analysis of TaTME cohorts showed a quality difference in low- versus high-volume centers, indicating a potential learning curve [2, 12].

The technical challenges and accompanying learning curve of the TaTME technique resulted in the off sprout of dedicated courses all over the world. In the Netherlands, a structured training pathway was set up in 2014 including a multiple step program of e-learning, didactic courses, detailed anatomy instruction, observation of a TaTME live procedure, a hands-on cadaver workshop, and the first cases proctored by TaTME experts. The current training pathway was set up to ensure adequate skills to participate in the COLORIII study, an international multicenter study evaluating the TaTME technique in terms of short- and long- term outcome powered upon oncological safety.

The aim of the present study was to capture the safety, clinical, and pathological outcomes of the implementation of TaTME in centers within the structured training pathway by collecting the data from the first ten patients in each participating center.

## Materials and methods

### Training pathway

The education and training pathway was set up in The Netherlands in 2014 as a structured program for postgraduate colorectal surgeons with known experience in laparoscopic TME surgery who had the intention to implement the TaTME technique in their center. In order to successfully introduce the TaTME technique, minimal pre-requisites were set out in order to enter the pathway. (1) Adequate skills and experience: prior training and experience in laparoscopic rectal cancer surgery with at least 50 laparoscopic TME cases. (2) Prior TAMIS experience: knowledge and skills of the transanal single port technique. (3) Case volume: the number of TaTME cases a year should be at least 20/year/center to ensure sufficient exposure of the entire team. (4) Adequate medical instruments including a continuous air insufflation system, an adequate transanal platform, and the possibility to perform a two-team approach.

This training program comprises a 2-day hands-on course in which a maximum of ten surgeons can participate enabling intensive interaction and individual tutoring. The course incorporates three different elements; didactic sessions, live TaTME surgery, and a hands-on training including box trainers and a cadaveric course. Didactic sessions included topics as theoretical background of TaTME, extensive pelvic anatomy by an anatomist, procedure-specific pitfalls (urethral injury, wrong plane of dissection e.g.,), patient selection, setup of surgical equipment, and step-by-step procedure training. Part of the education was available online (http://www.rectalcancersurgery.eu and http://www.iLappSurgery.com). The second part consisted of a multidisciplinary team (MDT) discussion of a case and a live TaTME case, performed at the Amsterdam University Medical Centers—location VUmc. Both surgeons and scrub nurses were invited as observers and technique was interactively demonstrated with informed consent of the patients.

The third part of the training was a full day hands-on training including box-training for the single-port technique skills and training the purse string. The final part was to practice an entire TaTME procedure on a cadaver in pairs, with experienced faculty providing help. At time of writing, over 200 surgeons from all over the globe have participated in one of our 22 courses since its launch in 2014.

The implementation of the technique in the center of the trainees was done by a structured clinical proctoring program until adequate proficiency was reached in agreement with the proctor to proceed alone. The group of proctors consists of eight surgeons who have each performed over 50 cases of TaTME and are trained in surgical education. Patients were informed to undergo a proctored TaTME. After each case, evaluation with the team was done. If requested by proctor or participating surgeon, an additional case of proctoring could occur in order to guarantee the quality as much as possible.

In October 2018, hospitals that have successfully completed the proctoring program were approached to share the data of the first ten TaTME procedures performed on patients with rectal cancer with curative intent. All of the 12 hospitals that were eligible to participate in our study agreed on participation.

### TaTME surgical procedure

The technique has been highly standardized as previously described [13]. First, the laparoscopic transabdominal phase starts with standard medial-to-lateral mobilization of the splenic flexure. Next, the patient is positioned in Trendelenburg and a medial-to-lateral approach to the sigmoid and rectum is performed, after the ligation of the superior artery including sigmoidal branches with preferably sparing of the left colic artery. Thereafter, the dissection is continued in the TME plane dorsally, both sides laterally and the beginning anteriorly. Identification of ureters and hypogastric plexus and nerve bundles has been mandatory. After ligation of the vessels, the transanal phase is started simultaneously with insertion of the transanal port and establishing pneumorectum with abdominal clamping of the distal sigmoid to avoid a pneumocolon. After closure with a purse string of the rectum below the tumor and ideal above the anorectal junction, the rectal tube is rinsed with povidone-iodine solution. After a full-thickness endoscopic transection of the rectum is achieved, the posterior TME plane, anterior plane, and both lateral planes are dissected, the latter with the help of abdominal retraction of the rectum. Anastomosis is preferably constructed with a circular stapling technique either side-to-end or end-to-end, specimen retraction is preferably done by a pfannenstiel incision.

### Patients

The Medical Ethics Review Board of the VU Medical Center in Amsterdam approved the study protocol and waived the need for informed consents. The first ten consecutive patients from each participating hospital with clinical suspicion of rectal cancer in whom a TaTME was performed with curative intent were eligible for this study. In October 2018, 12 hospitals completed our proctoring program and were all willing to participate in this study. All participating surgeons followed the structured TaTME training in the VU medical center prior to the proctoring program. Data were provided anonymously by the participating surgeon and checked by research assistants. No cases were excluded and all cases were consecutively. For the evaluated cases, all data were collected anonymously with entry as case numbers. In order to calculate the distance from the anal verge (AV) when only distance from anorectal junction was provided, the tumor distance measured from the anorectal junction was corrected by adding four centimeters for males and females [14–17]. The part of the rectum in which the tumor was situated was defined by distance from the AV as 0–6 cm, 7–11 cm, and 12–15 cm for low, mid, and high rectum, respectively [15].

### Outcomes

Primary outcomes were intraoperative complications. Secondary outcomes included operation time, conversion to laparotomy and postoperative complications, length of stay, and pathological outcomes (e.g., circumferential and distal resection margin, completeness of mesorectum according to classification of Quirke et al.). (18) In order to show a potential learning curve, outcomes of the first five procedures were compared to the outcomes of the sequential five procedures.

### Statistical analysis

A *p*-value ≦ 0.05 was considered statistically significant. For analysis of comparing results between the first and sequential five procedures, Chi-Square test (Fisher’s exact test when appropriate) in the case of categorical variables and Students T-test in the case of continuous variables were used. Mann–Whitney *U* test was used for continuous variables that were not normally distributed. Statistical analysis was performed using SPSS version 22 for Windows and Mac (SPSS, Chicago, Illinois, USA).

## Results

### Baseline characteristics

A total of 120 patients were included of which 53 operated with attending proctor and 67 without attending proctor. Baseline characteristics are shown in Table 1. The majority of patients were male (*n* = 91, 75.8%). The mean BMI was 26.9 kg/m^2^ (standard deviation (SD) 4.0) and age 65.4 years (SD 9.9). Tumors were located in the lower rectum in 45%, middle rectum in 46.7%, and upper rectum in 8.3% (Mean 6.9 cm from AV). Patients received either radiotherapy (*n* = 41, 34.2%), chemoradiotherapy (*n* = 36, 30.0%), or no neoadjuvant treatment (*n* = 43, 35.8%). The majority of tumors were classified as cT3 (73.7%) on MRI in the preoperative work-up. When comparing the first and sequential cohort of 60 patients, ASA classification was significantly higher in the second group (*p* = 0.021). Remaining baseline characteristics did not differ significantly.

**Table 1:** Patient characteristics

	Structured training program *n* = 120
Sex	
Male	91 (75.8)
Female	29 (24.2)
BMI (mean) (± SD)	26.9 (± 4.0)
Age (years) (mean) (± SD)	65.4 (± 9,9)
History of abdominal surgery	
No	91 (75.8)
Yes	29 (24.2)
History of transanal surgery	
No	115 (95.8)
Yes	5 (4.2)
ASA	
I	25 (21.4)
II	75 (64.1)
III	17 (14.5)
Missing data	3 (2.5)
Tumor height (AV) (cm) (mean) (± SD)	6.9 (± 3.1)
Tumor stage	
T1	6 (5.1)
T2	23 (19.5)
T3	87 (73.7)
T4	2 (1.7)
Missing data	2 (1.7)
Mesorectal fascia involvement	
No	97 (82.2)
Yes	21 (17.8)
Missing data	2 (1.7)
Preoperative therapy	
None	43 (35.8)
RT	41 (34.2)
CRT	36 (30.0)

Numbers in parentheses are percentages, unless mentioned otherwise

*BMI* body mass index (kg/m^2^), *SD* standard deviation, *ASA* American Society of Anesthesiologists, *cm* centimeters, *AV* anal verge, *RT* radiotherapy, *CRT* chemoradiotherapy

### Operative details

Table 2 shows the intraoperative outcomes of all patients. Transanal TME with primary anastomosis and diverting ileostomy was performed in 64.1% of patients (*n* = 77). In one patient, a transverse loop colostomy was performed due to clinical signs of obstruction prior to the TaTME. The most common anastomotic technique performed was mechanical stapling (94.9%) with a side-to-end or end-to-end anastomosis in 36.7% and 63.3% of patients, respectively. Mean operative time was 293 min (SD 93.4). Intraoperative complications were reported in six patients (4.9%).

**Table 2:** Operative details

	Structured training program *n* = 120
Type of surgery	
LAR	110 (91.7)
Intersphincteric	10 (8.3)
Anastomosis	
No	22 (18.3)
Yes	98 (81.7)
Stoma type	
None	20 (16.7)
Diverting ileostomy	77 (64.1)
Colostomy^a^	23 (19.2)
Technique anastomosis	
Hand sewn	5 (5.1)
Stapled	93 (94.9)
Type anastomosis	
Side-to-end	36 (36.7)
End-to-end	62 (63.3)
Specimen removal	
Pfannenstiel	68 (57.6)
Transanally	31 (26.3)
Stoma site	3 (2.5)
Laparotomy	5 (4.2)
Small transverse incision	11 (9.3)
Missing data	2 (1.7)
Operative time (min) (mean) (± SD)	293.0 (± 92.6)
Blood loss (ml) (median) (range)	100.0 (0–4050)
Conversion^b^	5 (4.2)
Intraoperative events	
Urethral injury	0 (0.0)
Pelvic bleeding	2 (1.7)
Rectal perforation	1 (0.8)
Small bowel injury	1 (0.8)
Ureter injury	1 (0.8)
Technical problems^c^	1 (0.8)

Numbers in parentheses are percentages, unless mentioned otherwise

*LAR* low anterior resection, *SD* standard deviation, *min* minutes, *ml* milliliters

^a^Includes 1 in situ loop transversostomy

^b^Includes 1 early conversion

^c^Complete unilateral dissection, unable to safely progress contralateral

Two of this six complications occurred during the transanal phase (1.7%); a rectal perforation and difficulty dissecting the right lateral plane resulting in completing the dissection laparoscopically. Two pelvic bleeding, iatrogenic injury to the small bowel and combined ureter and bladder injury occurred in four patients during the laparoscopic phase (3.3%). In 5 out of 120 cases (4.2%) conversion to laparotomy was necessary, due to portal hypertension, a combined ureter and bladder injury, difficulties due to a BMI of 40 in combination with a small male pelvis and difficulty mobilizing the splenic flexure during the laparoscopic phase. The fifth conversion was done due to a difficult transanal phase; an inability to find a safe lateral plane due to a combination of a curved anatomy and the initial transanal unilateral dissection with connection to the peritoneal cavity. The first four conversions occurred during the abdominal phase and without attending proctor, the latter with attending proctor. In summary, intraoperative complications occurred twice during the abdominal phase (1.7%), and in four cases (3.3%) during the transanal phase. For the non-converted cases, specimen removal was mostly performed through a pfannenstiel incision (57.6%) and secondly by transanal extraction (26.3%).

### Postoperative outcomes

Median hospital stay was reported as 7 days (range 3–43) (Table 3). Overall postoperative morbidity rate was 45.0% (*n* = 54). Major complications within 30 days, graded as Clavien–Dindo IIIa or IIIb were seen in 19.2% (*n* = 23) of patients. No Clavien–Dindo gr IV or V complications were reported. A primary anastomosis was performed in 81.7% of the cases (*n* = 98) of whom 17 patients (17.3%) encountered anastomotic problems. Treatment in these cases occurred by colostomy (4), diverting ileostomy (4), resuture (2), drainage (1), novel anastomosis (1), and endosponge (vacuum therapy) (5). Four patients with a primary end colostomy developed a presacral abscess despite the absence of an anastomosis which was treated by transanal drainage (2), CT-guided drainage (1), and endosponge (1). Overall, twenty patients had anastomotic problems or presacral abscesses of which 81% (*n* = 17) were seen in patients with primary anastomosis (Table 4).

**Table 3:** Postoperative details

	Structured training program *n* = 120
Hospital stay (days) (median) (range)	7 (3–43)
Postoperative complications CD	
None	66 (55.0)
Minor (CD I-II)	31 (25.8)
Major (CD ≥ III)	23 (19.2)
IIIa	7 (30.4)
IIIb	16 (69.6)
Anastomotic leakage < 30 days^a^	17 (17.3)
Anastomotic treatment^a^	
Endosponge	5 (5.1)
Temporary ileostomy	4 (4.1)
Unintended colostomy	4 (4.1)
Suture	2 (2.0)
Drainage	1 (1.0)
Novel anastomosis	1 (1.0)
30-day mortality	0 (0.0)

Numbers in parentheses are percentages, unless mentioned otherwise

*CD* Clavien–Dindo classification

^a^Only patients selected with anastomosis (*n* = 98)

**Table 4:** Pathology reports

	Structured training program *n* = 120
Pathology stage	
pT0	9 (7.6)
pT1	16 (13.6)
pT2	32 (28.8)
pT3	59 (50.0)
n.a	2 (1.7)
Quality of specimen (Quirke)^a^	
Complete	107 (89.2)
Nearly complete	13 (10.8)
Incomplete	0 (0.0)
CRM involvement	6 (5.0)
DRM involvement^b^	0 (0.0)
Lymph nodes harvested (mean) (± SD)	17.0 (± 7.2)
Lymph nodes positive (median) (range)	0 (0–7)

Numbers in parentheses are percentages unless mentioned otherwise

*CRM* circumferential resection margin, *DRM* distal resection margin, *SD* standard deviation

^a^From Quirke et al. [18]

^b^1 missing patient

### Pathologic outcomes

The quality of specimens according to Quirke’s classification was complete or nearly complete in all patients (89.2% and 10.8%, respectively). A positive circumferential resection margin (tumor invasion < 1 mm from non-peritonealized surface of the rectum) was reported in 5.0% of patients. Distal resection margins were negative (> 1 mm) in all 120 patients.

### Short-term training

No significant differences in operative time between the first cohort of proctored and second cohort of unproctored five procedures per center were found (283.6 and 302.5 min, respectively, *p* = 0.266). (Table 5) In both groups, three intraoperative adverse events occurred suggesting no major increase in intraoperative difficulties resulting in visceral injuries or an increase in conversion rate. A complete specimen (Quirke) was excised more frequently in the second cohort which was a significant difference; 80.0% versus 98.3% for procedure 1–5 and 6–10, respectively (*p* = 0.001). The circumferential resection margin involvement was slightly higher in the second half of procedures (1.7% versus 8.3%) but this did not reach significance. Postoperative morbidity was equal for the first and second cohort as can be seen in Table 5. Severe short-term morbidity, defined as Clavien–Dindo ≥ III, was equally distributed. Moreover, anastomotic problems were encountered in 9 of 48 and 8 of 50 cases, respectively.

**Table 5:** Learning effect within structured training program

	Patients 1–5 (60)	Patients 5–10 (60)	*p*-value
Sex (male)	45 (75.0)	46 (76.7)	0.831
BMI (mean) (± SD)	26.5 (± 3.5)	27.3 (± 4.5)	0.269
Age (years) (mean) (± SD)	64.9 (± 10,3)	66.0 (± 9.5)	0.540
ASA			
I	15 (26.3)	10 (16.7)	0.017
II	39 (68.4)	36 (60.0)	
III	3 (5.3)	14 (23.3)	
Tumor height (AV) (cm) (mean) (± SD)	6.7 (3.1)	7.1 (3.0)	0.519
Tumor stage			
T1	3 (5.1)	3 (5.1)	1000*
T2	12 (20.3)	11 (18.6)	
T3	43 (72.9)	44 (74.6)	
T4	1 (1.7)	1 (1.7)	
Mesorectal fascia involvement			
No	49 (83.1)	48 (81.4)	0.810
Yes	10 (16.9)	11 (18.6)	
Preoperative therapy			
None	18 (30.0)	25 (41.7)	0.406
RT	22 (36.7)	19 (31.6)	
CRT	20 (33.3)	16 (26.7)	
Type of surgery			
LAR	53 (88.3)	57 (95.0)	0.186
Intersphincteric	7 (11.7)	3 (5.0)	
Operative time (min) (mean) (± SD)	283.6 (± 80.1)	302.5 (± 103.6)	0.266
Conversion	1 (1.7)	4 (6.8)	0.207*
Intraoperative complications	3 (5.0)	3 (5.1)	0.1000*
Hospital stay (days) (median) (range)	8 (3–43)	7 (3–25)	0.521*
Postoperative complications CD			
None	31 (51.7)	35 (58.3)	0.750
Minor (CD I-II)	17 (28.3)	14 (23.4)	
Major (CD ≥ III)	12 (20.0)	11 (18.3)	
Anastomotic leakage < 30 days^a^	9 (18.8)	8 (16.0)	0.719
Quality of specimen (Quirke)^b^			
Complete	48 (80.0)	59 (98.3)	0.001
Nearly complete	12 (20.0)	1 (1.7)	
Incomplete	0 (0.0)	0 (0.0)	
CRM involvement	1 (1.7)	5 (8.3)	0.207*

Numbers in parentheses are percentages, unless mentioned otherwise

*BMI* Body Mass Index (kg/m^2^), *SD* standard deviation, *ASA* American Society of Anesthesiologists, *RT* radiotherapy, *CRT* chemoradiotherapy, *LAR* low anterior resection, *min* minutes, *CD* Clavien–Dindo classification, *CRM* circumferential resection margin

*Fisher’s Exact Test or Fishers–Freeman–Halton Test or Mann–Whitney *U* Test

^a^Only patients selected with anastomosis (*n* = 98)

^b^From Quirke et al. [18]

## Discussion

This study shows the outcome data during the initial phase of a structured TaTME training and proctoring curriculum in the Netherlands in 12 centers, and within each center the first ten patients resulting in a total of 120 patients. We have demonstrated that the implementation within a structured training pathway is relatively safe with a low rate of intraoperative complications of 4.9% and good quality of resection and specimen. The resection quality as addressed by pathology showed that complete or nearly complete specimen was obtained in 100%, similar to negative distal resection margin. Circumferential resection margin was positive in 5.0% of patients. Postoperative complications according to Clavien–Dindo ≥ III occurred in 19.2%. These results suggest that the structured training pathway does result in safe introduction of the technique without major intraoperative complications and safe results comparable to those obtained from laparoscopic TME surgery [8, 19].

Compared to the international TaTME registry, especially the intraoperative event rate seems low; the registry reports 30.6% intraoperative adverse events rate, of which a vast majority (18%) regarded technical problems leaving 12.6% to visceral injuries (1.8%) of which 12 urethral injuries (0.8%), incorrect dissection planes (5.7%), and pelvic haemorrhage (4.2%) [9]. Concern exists for underreporting since publications bias of cohort studies is present and rumors about intraoperative complications as urethral injury, pelvic sidewall injury, rectal tube perforation, and venous CO_2_ embolisms are discussed at conferences and courses frequently but fail to be represented in the available literature. The proctor-guided implementation of TaTME in the current series showed that intraoperative complications were encountered in 5.0% and conversion to laparotomy was necessary in 4.2%, suggesting that a proctor-based introduction potentially lowers the frequency of intraoperative difficulties and adverse events. To illustrate this, no injuries to the urethra (0%) occurred and visceral injury was encountered in 3 of 120 cases (2.5%). An incorrect dissection plane was reported in one patient (0.8%) which is considerably lower than the 5.7% of the previously mentioned registry. Abbott et al. [20] recently published the results of implementing TaTME Australia and New Zealand using a training pathway which includes on-site proctoring and showed a safe introduction with low conversion rate (3%), no intraoperative visceral complications but did report two rectal wall perforations.

Postoperative morbidity was reported as 45% overall complications, including 19.2% major complication and 17.3% leakage rate. Although the structured training seems to provide intraoperative safety the postoperative event rate is high. Several reasons may account for this. First, all pelvic abscess and subclinical leaks were included and regarded as a anastomotic leakage. Second this is audited cohort data from multicenter cohort within a learning curve. Most likely the learning curve is associated with prolonged operating time and bacterial spill may negatively influence morbidity rate [12, 21]. It is shown that the learning curve of TaTME is associated with an increase in major surgical postoperative complications (Clavien–Dindo ≥ III) in the first forty cases [12]. In this study, the anastomotic leak rate was 27.5% for the first 40 cases and decreased to 5% for the next forty cases. In addition, the international registry data also show a relative high anastomotic leak rate, 15.7% [9, 12]. Similar leakage rates have been reported by the Dutch national audit (90 days; 20%) [9, 22–24]. The delayed leak and/or presacral abscess potentially may come as a consequence of the open rectal stump which raises a concern for bacterial contamination as demonstrated by Velthuis et al. but needs further prospective investigation [21]. Third reason for the experienced relative high morbidity is the selected patient group with 45% distal tumors and 64.2% was treated with neoadjuvant therapy. These distal tumor resections are more prone to morbidity.

Within the included centers, no difference in intraoperative complications, postoperative morbidity, or anastomotic leak rate was present when comparing the first proctored cohort of five to the second cohort of non-proctored patients. This suggests that the learning effect was not present for adverse outcomes in our training program and that therefore five cases seem efficient. However, evaluating a surgical learning curve needs higher number of cases (80) to allow a CUSUM analysis [12, 25–27]. Future studies will address learning curves within centers that have undergone a structured training curriculum [2, 8, 28, 29]. In laparoscopic surgery, learning curve ranging from 100 to 150 cases as self-taught learning curve and 40–60 with proctoring/teaching programs is reported [25, 28–30]. The UK LapCo training program showed the safe widespread implementation of supervised training for laparoscopic colorectal surgery [31]. Within this program, outcomes between experienced consultant trainers and trainees were similar regarding adverse events [31, 32]. In addition, a meta-analysis of Kelly et al. [33] on 19 studies reporting a total of 14.344 colorectal resections showed no significant increase in anastomotic leak rate, conversion, or worse pathological outcomes in procedures performed by trainees.

In our series, the primary end colostomy percentage of 18.8 is lower than the 34% in COLOR II [23]. However, compared to the 9% colostomy rate from the TaTME registry this seems a high rate [11]. Transanal total mesorectal excision possibly enables an increase of the creation of an anastomosis in lower rectal cancer which previously would have been subject for end colostomy. In further detail, this (very) low colorectal or coloanal anastomosis might be suitable for patients without the oncologic necessity for an abdominoperineal resection but in whom it is technically impossible to create an anastomosis by only transabdominal laparoscopy. For this category, the preoperative function of the anal sphincter is important in the consideration whether to make an anastomosis or an end colostomy. This can probably be attributed to patient selection in the beginning of implementation in the Netherlands, as surgeons try to avoid major APE surgery for patients and select low rectal cancer for TaTME in order to achieve a primary anastomosis.

Long-term oncological outcomes in terms of local recurrences have to be awaited due to the limited duration of follow-up in this series. As CRM involvement seems to be a strong predictor of local recurrence, the encountered 5.0% is lower compared to 6.3–12.1% as reported by the recent Laparoscopic TME RCTs. (Table 6) [8, 10, 23, 34–37]. The quality of the specimen as defined by Quirke et al. was incomplete in 0 out of 120 cases, with a significant increase of a complete specimen when comparing the proctored cohort versus the non-proctored cohort patients: 80% versus 93.3%, respectively (*p* = 0.01) [18, 34]. A non-significant difference of CRM involvement was observed (1.7% vs 8.3% *p* = 0.207). This increase of CRM while oppositely improving the quality of specimen might be attributed to tumor characteristics which were not captured in this study. It might be contributed to a higher proportion of anterior located tumors where, due to tapering of the mesorectum towards the pelvic floor, the bowel wall is directly adjacent to the mesorectal fascia without any intermediary mesorectal fat. Distal resection margin was free in all cases suggesting a high rate of R0 resection in our series.

**Table 6:** Structured training program compared to other studies

	Structured training program *n* = 120	High volume *n* ≥ 30 cohorts *n* = 478^a^	TaTME registry *n* = 1594*	Lap TME trials *n* = 1411^b^
Sex (male)	75.8	67.4	67.8	65
Age (years) (mean)	65.4	63.8	63.7	64
BMI (mean)	26.9	26.1	26.3	26.1–27
Neoadjuvant (c)RT	64.2	73.0	56.1	61.9
Tumor height (cm) (mean) (AV)	6.9	6.5	4.0^$^	NA
cT3 or cT4	75.4	69.3	69.0	NA
Conversion	4.2	2.7	5.6	13.0
Anastomotic leakage	17.3	NA	15.7	7.9
pT3 or pT4	50.0	45.1	43.5	NA
Quality of specimen (Quirke)^d^				
Complete	89.2	89.7	85.8	87.0
Nearly complete	10.8	9.0	10.8	13.0
Incomplete	0	1.3	3.4	4.0
Missing			9.7	6.0
CRM involvement	5.0	4.5	4.1	8.0
DRM involvement	0.0	NA	0.7	NA

All numbers are percentages, unless mentioned otherwise

*TaTME* transanal total mesorectal excision, *Lap TME* laparoscopic total mesorectal excision, *BMI* body mass index (kg/m^2^), *(c)RT* (chemo)radiotherapy, *cm* centimeters, *AV* anal verge, *CRM* circumferential resection margin, *DRM* distal resection margin

*From Penna et al. [9]

^a^From Deijen et al. [2]

^b^From van Oostendorp et al. [8]

^c^Median from anorectal junction

^d^From Quirke et al. [18]

Some limitations of this study should be mentioned; the small sample size and the lack of more than ten patients per center. Nevertheless, the main purpose of this study was to show the feasibility and safety of the training and proctoring program; the low intraoperative complications and promising pathological outcomes indicate that competency to safely perform a TaTME is achieved. By extending the cohort to larger numbers per center, too many inclusions could therefore be seen as unethical, especially in case if we had encountered major intraoperative complications or R1 resections in the second half of the patients. We do feel that additional audit, quality control, and potentially extension of the amount of procedures with a proctor should be deliberated in future series. At time of data accrual, all centers who completed the program have participated in this study.

## Conclusion

This study describes the safe introduction of TaTME in the Netherlands within a structured training program deemed necessary due to the high complexity of this novel surgical approach. Intraoperative complication rate was low and TaTME specific complications such as pelvic sidewall injury and urethral transection occurred rarely. However, postoperative morbidity and anastomotic leak rate emphasize the need for careful implementation and need for randomized data as well as long-term outcomes on local recurrences.
